# Biocompatible OFETs for Selective and Real-Time Bacterial Detection Using BSA and Lysozyme Layers

**DOI:** 10.1021/acsabm.4c01618

**Published:** 2025-04-03

**Authors:** Po-Hsiang Fang, Guan-Xu Chen, Shuying Wang, Ching-Hao Teng, Wen-Chun Huang, Horng-Long Cheng, Wei-Yang Chou

**Affiliations:** † Department of Photonics, National Cheng Kung University, Tainan 70101, Taiwan; ‡ Department of Microbiology and Immunology, Institute of Basic Medical Sciences, College of Medicine, National Cheng Kung University, Tainan 70101, Taiwan; § Institute of Molecular Medicine, National Cheng Kung University, Tainan 70101, Taiwan

**Keywords:** organic field-effect transistors (OFETs), bacterial sensing, gram-positive and gram-negative bacteria, protein-based materials, real-time monitoring, spike measurement

## Abstract

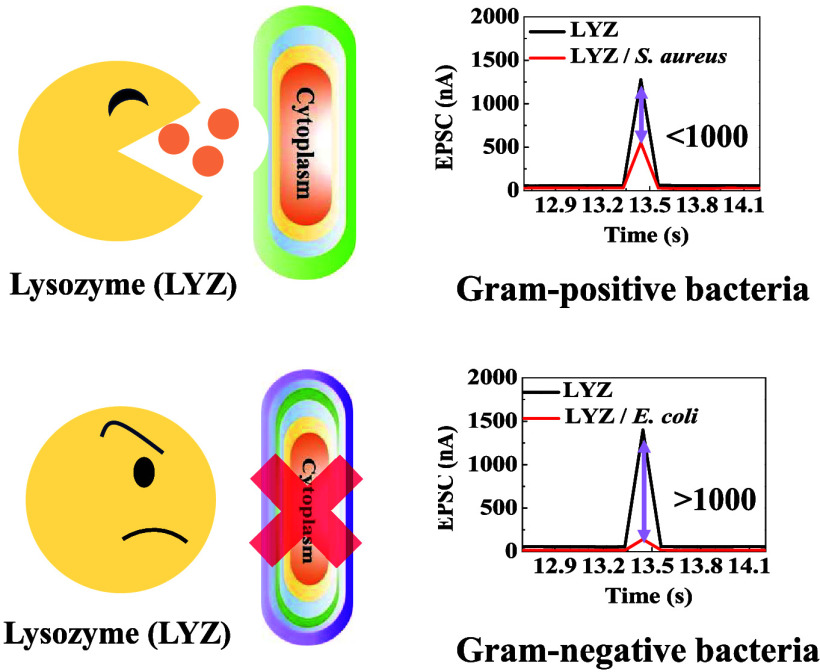

In the realms of modern medicine and environmental monitoring, there is an escalating demand for bacterial detection technologies that are rapid, precise, and highly sensitive. Conventional methods, however, are often hindered by their time-intensive nature, procedural complexity, and reliance on specialized laboratory equipment. This study introduces an innovative approach utilizing bovine serum albumin (BSA) as the dielectric layer and lysozyme (LYZ) as the bacterial sensing layer in organic field-effect transistors (OFETs). The combination of BSA and LYZ enhances both biocompatibility and detection sensitivity, enabling precise differentiation between Gram-positive and Gram-negative bacteria. BSA not only stabilizes the electrical performance of the OFET but also offers biodegradability and water solubility, contributing to environmental sustainability. These biocompatible OFETs can accurately detect bacterial concentrations ranging from 10^4^ to 10^8^ CFU/mL, with real-time response capabilities via multispike measurements. This research represents a significant step forward in the development of advanced, portable biosensors for use in complex biological environments, advancing bacterial detection technology.

## Introduction

In recent years, the development of biosensors has gained significant attention in the healthcare sector, primarily aimed at addressing various diseases and chronic conditions associated with modern lifestyles. These health issues have become global societal challenges, substantially impacting public health and quality of life.^1−5^ Consequently, the need for accurate and rapid biomarker detection technologies has become a crucial focus in biosensor research. Traditional biomolecule detection methods, such as polymerase chain reaction (PCR) and enzyme-linked immunosorbent assay (ELISA), offer notable advantages in sensitivity and specificity. However, these techniques typically require large, expensive instrumentation, leading to higher detection costs and prolonged experimental procedures, thus complicating early diagnosis for patients.

In contrast, research on organic semiconductor materials has advanced rapidly, particularly in fields such as flexible electronics, organic field-effect transistors (OFETs), organic light-emitting diodes (OLEDs), and organic solar cells.^6−9^ Among these, OFETs have found widespread application. The mechanical flexibility of OFETs allows them to adapt to various shapes and surfaces, making them particularly suitable for wearable devices.^10−13^ Additionally, OFETs are cost-effective to manufacture and exhibit excellent biocompatibility, making them ideal for biological applications.^14,15^ This biocompatibility enables OFETs to interact with biological samples, identifying specific biomolecules by analyzing changes in electrical signals, thereby achieving biorecognition functionality.^16−18^

Biomaterials, such as DNA, carbohydrates, peptides, and proteins, are naturally abundant and require no additional chemical synthesis, making them highly promising for developing environmentally friendly electronic devices. Their biodegradability, bioresorbability, and biocompatibility have led to the widespread use of biomaterials in OFET fabrication.^19,20^ Proteins, in particular, can function as dielectric layers due to their insulating properties.^21,22^ Commonly used proteins for this purpose include silk fibroin, bovine serum albumin (BSA), collagen, and gelatin.^23,24^ BSA, a natural protein with a molecular weight of approximately 66,500 Da, is highly concentrated, water-soluble, and stable.^25^ Due to its excellent biocompatibility, BSA is widely used in immunology, cell culture, and enzyme activity studies.^26,27^ Its insulating properties and biodegradability make it an excellent candidate for use as a dielectric material in OFETs, enhancing device performance and detection specificity.

Gan et al. demonstrated the application of BSA as a dielectric layer in α-IGZO thin-film transistors, reporting high drain current at a gate voltage as low as 2 V and excellent field-effect mobility, with stable performance over a 60-day period.^28^ Similarly, Hwang et al. used BSA as a dielectric layer with pentacene as the semiconductor layer, showing that BSA functions like a polymer electrolyte double-layer capacitor (EDLC), significantly enhancing device performance. In atmospheric conditions, the field-effect mobility reached 4.7 cm^2^/V·s, illustrating BSA’s potential to improve thin-film transistor performance.^29,30^

Lysozyme (LYZ), a 129-amino-acid antimicrobial protein often referred to as the “body’s own antibiotic,” has wide applications in the food industry for preserving meat and dairy products.^31^ At neutral pH, LYZ carries a positive charge, which contributes to its unique properties.^32^ Beyond food preservation, LYZ also serves as a biomarker for various diseases, including breast cancer, Alzheimer’s disease, and rheumatoid arthritis.^33^ Recent studies have shown that LYZ can be used to detect the pH of aqueous solutions and exhibits enhanced sensitivity to specific biomolecule expressions. These findings highlight LYZ’s potential for biosensing applications, providing a strong foundation for the development of advanced protein-based biosensors.

The use of biomaterials in OFETs simplifies manufacturing processes, reduces costs, and improves device performance. Proteins, serving as dielectric layers in the layered structure of OFETs, offer insulating properties and biodegradability, delivering performance characteristics that are difficult to achieve with traditional organic or inorganic materials. OFETs, with their high sensitivity, can convert and amplify biological signals. By employing specific binding interactions between recognition elements and target analytes, OFETs achieve highly selective detection. The combination of sensitivity, selectivity, and biomaterial integration positions OFETs as promising tools for next-generation biosensing applications.

In this study, we employed BSA and LYZ as the gate dielectric layer and bacterial sensing layer, respectively, with N, N’-Ditridecylperylene-3,4,9,10-tetra-carboxylic diimide (PTCDI-C_13_) as the active layer in our OFETs. We focused on the application of the LYZ sensing layer in OFET devices and its impact on bacterial detection. LYZ was spin-coated onto the semiconductor channel, and we compared devices with and without the LYZ sensing layer. We tested Gram-negative bacteria, including *Escherichia coli* (*E. coli*), *Klebsiella pneumoniae* (*Kp*), and *Pseudomonas aeruginosa* (*P. aeruginosa*), as well as Gram-positive bacteria, such as *Staphylococcus aureus* (*S. aureus*) and *Bacillus subtilis* (*B. subtilis*), at a concentration of 10^8^ CFU/mL, on the LYZ-coated *n*-type OFET channel.

Traditional bacterial detection techniques, while highly sensitive, often rely on expensive instrumentation and complex procedures, limiting their applicability for on-site detection and real-time diagnosis. Rapid differentiation between Gram-positive and Gram-negative bacteria is critical for guiding antibiotic treatment strategies and infection control. However, existing rapid detection methods still face significant challenges in terms of selectivity and sensitivity. Therefore, developing an efficient, sensitive, and selective bacterial detection platform has become a pressing research goal. Current literature rarely explores the use of LYZ as a bacterial sensing layer, yet its ability to disrupt the cell walls of Gram-positive bacteria offers promising potential for targeted detection.

To address this, we propose a pioneering bacterial detection method that integrates LYZ with OFET technology, aiming to develop a high-sensitivity, high-selectivity detection platform and investigate its applications in biomedical fields. Notably, the use of LYZ as a bacterial sensing layer has been minimally explored, underscoring the innovation of our approach. We leverage the thicker peptidoglycan layer of Gram-positive bacteria and analyze the electrical and synaptic behavioral changes induced by LYZ-mediated cell wall disruption. This method not only achieves specific detection of Gram-positive bacteria but also effectively distinguishes between Gram-positive and Gram-negative bacteria, demonstrating the high efficiency and application potential of the device in bacterial identification.

## Experimental and Methods

### Device Fabrication and Characterization

First, the glass substrate was cut to dimensions of 2 cm × 1.6 cm, then immersed in a cleaning solution and scrubbed with cotton swabs to remove surface contaminants. The substrate was subsequently cleaned with acetone and isopropanol, followed by baking in an oven at 80 °C for over 4 h. After cleaning, an 80 nm thick Al film was thermally deposited onto the glass substrate, serving as the gate electrode, which was patterned using a shadow mask. The Al gate electrode was then treated with oxygen plasma at 150 W for 15 s to form a thin AlO*_x_* layer with a high dielectric constant.

**Figure 1:**
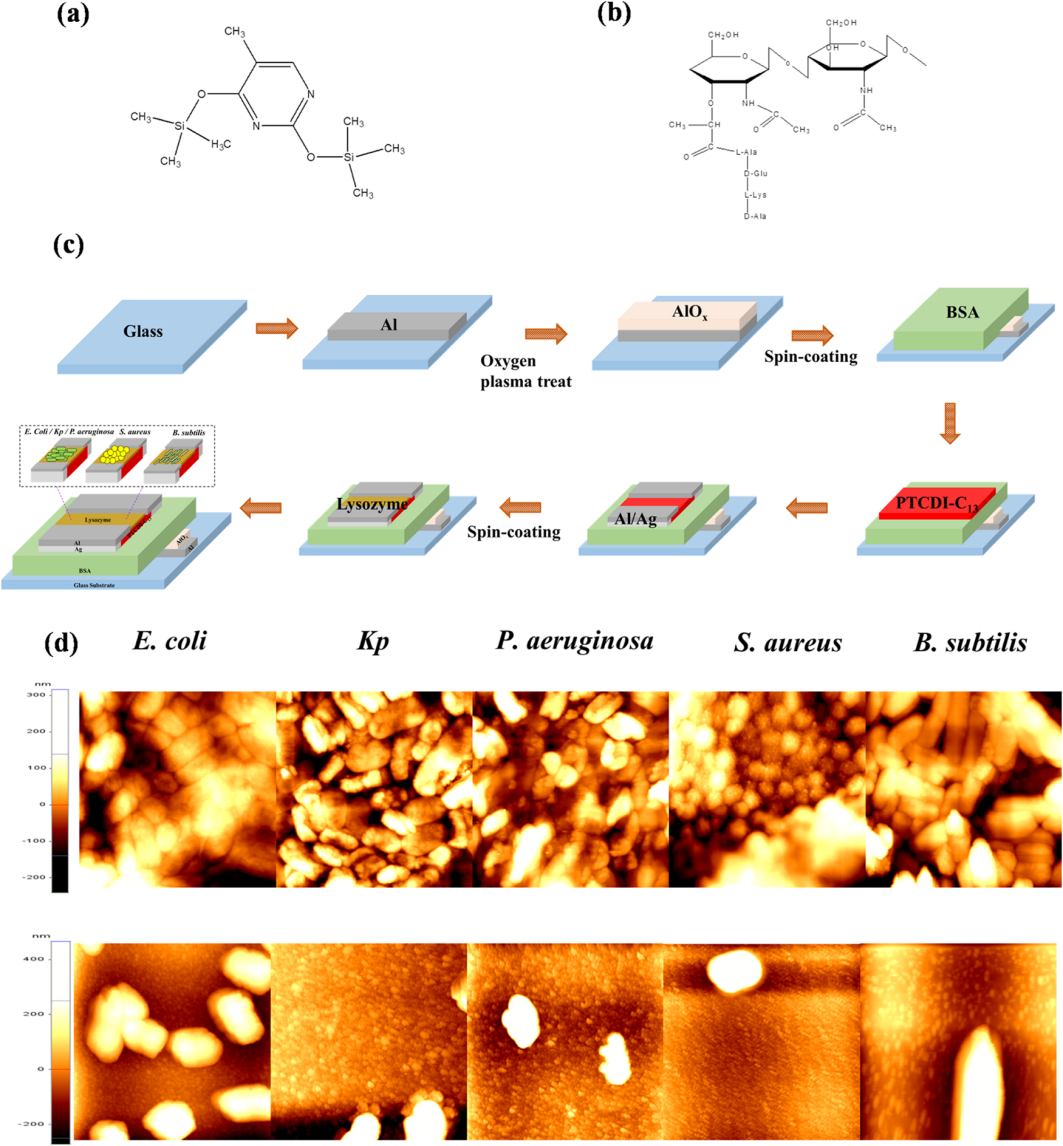
Protein material structures: (a) BSA, (b) LYZ. (c) Flowchart of device fabrication for an organic field-effect transistor-based biosensor; (d) AFM images of Gram-negative and Gram-positive bacteria dropped onto PTCDI-C_13_ film (top row) and LYZ sensing layer (bottom row).

BSA powder was dissolved in ultrapure water, and 1 μL of the solution was measured using a Protein A280 Nanodrop 2000 spectrophotometer to determine its concentration. The BSA concentration for this experiment was 9 mg/mL. The solution was filtered and purified to extend its shelf life. Spin coating was then used to apply the BSA solution onto the substrate, forming a dielectric modification layer. The chemical structure of BSA is shown in Figure 1a. The detailed molecular structure can be found in Supporting Information Figure S1a.

Next, a metal mask was used to define the pattern for the PTCDI-C_13_ semiconductor, and thermal evaporation was used to deposit a 60 nm thick semiconductor layer. The detailed chemical structure can be found in Supporting Information Figure S1b. After the active layers were deposited, an 80 nm thick Al/Ag composite film, serving as the source and drain electrodes, was deposited through a shadow mask. The channel length (*L*) and width (*W*) for the *n*-channel OFETs were 100 and 2000 μm, respectively.

LYZ was prepared at a concentration of 9 mg/mL, and its chemical structure is shown in Figure 1b. The preparation method for LYZ was the same as for BSA, with physical absorption onto the semiconductor channel, followed by spin coating and drying for 2 h to complete the preparation of the sensing layer. The fabrication flowchart of the device is shown in Figure 1c. The molecular structure of LYZ is shown in Supporting Information Figure S1c.

The electrical characteristics of the OFETs were measured using a Keithley 4200 semiconductor characterization system inside a nitrogen-filled glovebox. Surface morphology of the thin films was examined using atomic force microscopy (AFM, Park XE-100).

### Cell Culture

For bacterial testing, three Gram-negative Bacteria *E. coli*, *Kp*, *P. aeruginosa* and two Gram-positive Bacteria *S. aureus*, *B. subtilis*, whose shapes were shown on top panel of Figure 1d, were used as test strains. All bacteria were cultured in Luria–Bertani (LB) medium at 37 °C with shaking at 200 rpm for 24 h. Following incubation, the bacterial cultures were centrifuged at 8500 rpm for 5 min to pellet the bacteria. The culture medium was discarded, and the bacteria were resuspended in 1000 μL of deionized water. The bacterial solutions were serially diluted to concentrations ranging from 10^4^ to 10^8^ CFU/mL. Approximately 100 μL of each diluted sample was evenly spread on agar plates specific to each strain, and the plates were incubated overnight at 37 °C to allow for bacterial colony formation.

## Results and Discussion

### Electrical Characterization and Synaptic Response of LYZ-Modified OFETs

We employed AlO_*x*_ and BSA with different concentrations as the dielectric layers for the devices, with concentrations of 9, 30 mg/mL, and 100 mg/mL, corresponding to film thicknesses of 60, 300, and 1000 nm, respectively. In devices using 9 mg/mL BSA as the dielectric layer, the simulated synaptic behavior demonstrated a rapid increase in drain current upon applying a single stimulus, followed by a gradual decay to a stable state after the stimulus was removed. During this process, the signal-to-noise ratio (SNR) reached up to 3 orders of magnitude, indicating a significant distinction between the postsynaptic current and the background current.

In contrast, devices with 30 or 100 mg/mL BSA as the dielectric layer exhibited lower postsynaptic current amplitudes and a significantly reduced SNR, making it difficult to clearly differentiate the signal from the background current (as shown in Supporting Information Figure S2). Furthermore, devices using AlO_*x*_ as the dielectric layer did not exhibit noticeable postsynaptic current responses.

From the analysis of the transfer curves, it can be observed that when the dielectric layer is composed of AlO_*x*_, although the saturation current reaches 10^–6^ A, the leakage current is as high as 10^–7^A, indicating inadequate insulation, which increases the likelihood of device breakdown during operation. However, when BSA is introduced into the AlO_*x*_ dielectric layer, the insulation performance of the device improves significantly, with the leakage current reduced to the range of 10^–10^ to 10^–12^ A. Notably, the dielectric layer containing 9 mg/mL of BSA exhibits excellent switching characteristics, demonstrating a favorable subthreshold swing (*S.S.*) and an on/off current ratio exceeding 10^5^. As the BSA concentration increases further, both the *S.S.* and saturation current decrease, primarily due to the effect of dielectric thickness on the electric field, as shown in Supporting Information Figure S3.

These results collectively indicate that a BSA concentration of 9 mg/mL represents the optimal condition for the dielectric layer, effectively supporting stable and distinct synaptic behavior in the devices while providing excellent signal differentiation capability.

**Figure 2:**
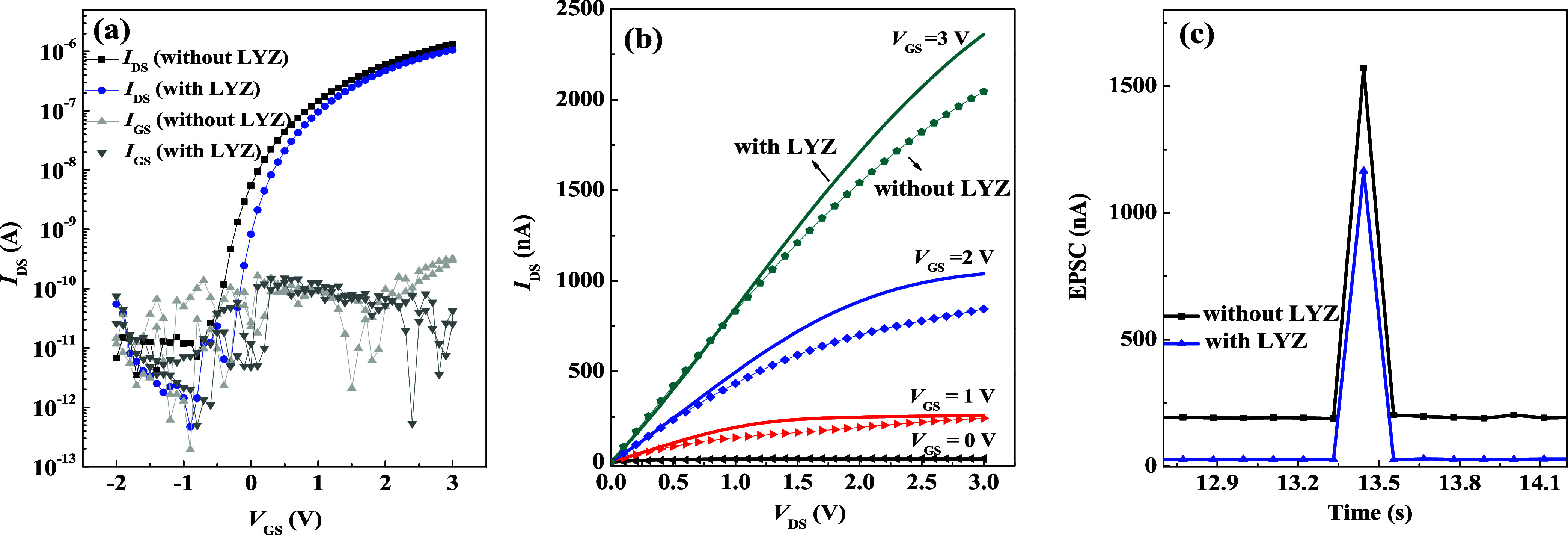
Electrical characteristics of OFETs with and without LYZ: (a) transfer curves; (b) output curves; (c) postsynaptic currents.

In Figure 2, we further investigate the impact of adding the LYZ sensing layer on the electrical properties of the device. From the transfer and output curve measurements shown in Figure 2a,b, it is evident that the device with the LYZ sensing layer exhibits a slight decrease in saturation current. This can be attributed to the oxygen plasma treatment of the PTCDI-C_13_ semiconductor layer prior to spin-coating the LYZ, which significantly affects the electrical characteristics of the thin film. Oxygen plasma alters the surface properties of the organic semiconductor, while water molecules in the LYZ solution may penetrate the semiconductor surface, leading to a reduction in the saturation current.

Further analysis reveals that the threshold voltage (*V*_TH_) shifted from −1 mV in the device without the LYZ sensing layer to 80 mV after its addition, indicating a positive shift. Literature suggests that LYZ carries a positive charge, which primarily affects electrons—the majority carriers in *n*-type semiconductors.^32,34^ The positive charges in LYZ attract electrons in the channel, necessitating a larger positive voltage to form the current channel, thereby causing the threshold voltage to approach zero volts. However, the *S.S.* increased slightly from 0.24 to 0.26 V/dec, indicating minor degradation in performance. This degradation is likely due to water molecules disrupting the interface between the semiconductor and dielectric layers, reducing gate control over the device channel and affecting the overall electrical performance. A summary of these electrical parameters is provided in Table 1.

**Table 1:** Electrical Parameters of OFETs, Including Dielectric Capacitance, Threshold Voltage, Subthreshold Swing, On/Off Current Ratio, and Field-Effect Mobility, with and without LYZ

	*C* (1000 Hz)	*V*_TH_ (mV)	*S.S.* (V/dec)	on/off ratio	μ (cm^2^ V^–1^ s^–1^)
without LYZ	1.71 × 10^–7^	–1	0.24	1.85 × 10^5^	8.71 × 10^–2^
with LYZ		80	0.26	2.03 × 10^5^	7.43 × 10^–2^

To further investigate the synaptic behavior of these devices, single-spike measurements were performed on both devices, as shown in Figure 2c. The experiments were conducted with an “On” time of 50 ms, “Off” time of 5 ms, and a base voltage of 3 V. The results indicate a significant decrease in excitatory postsynaptic current (EPSC) performance after the addition of the LYZ sensing layer, which is consistent with the observations from the transfer curves. This suggests that the presence of LYZ adversely affects current conduction properties, thereby influencing synaptic behavior. Despite the decrease in EPSC, the value remained above 1000 nA, indicating that the devices retained a degree of stability.

### Investigation of Synaptic Responses to Gram-Negative and Gram-Positive Bacteria in LYZ-Modified Devices

**Figure 3:**
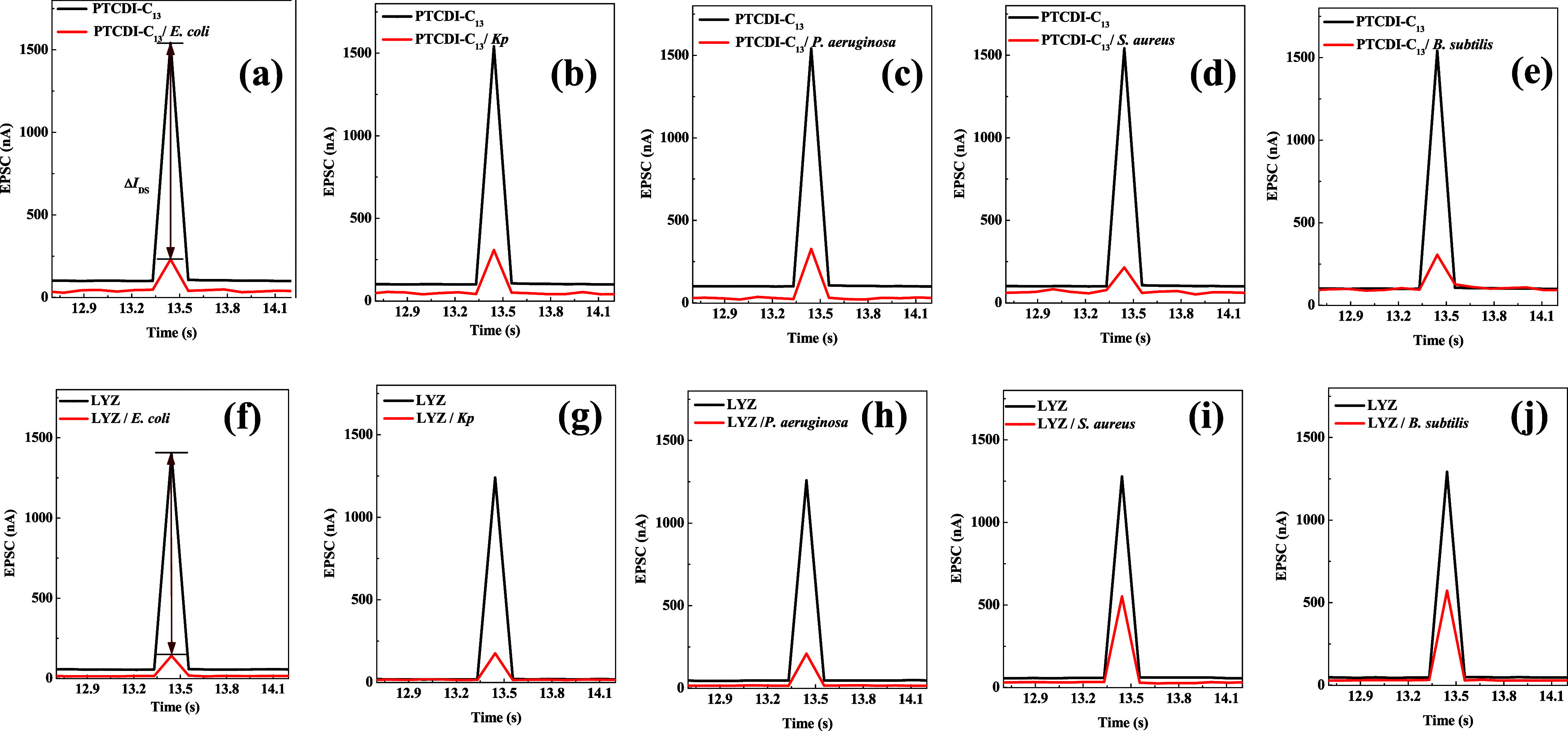
Postsynaptic current graphs for the PTCDI-C_13_ device with and without the LYZ sensing layer. (a) and (f) *E. coli*; (b) and (g) *Kp*; (c) and (h) *P. aeruginosa*; (d) and (i) *S. aureus*; (e) and (j) *B. subtilis*.

To investigate the effects of different bacteria on devices with a LYZ sensing layer, we applied 1 μL solutions of both Gram-negative and Gram-positive bacteria onto the sensing layer containing LYZ on the PTCDI-C_13_ device channel, each at a concentration of 10^8^ CFU/mL. We observed the changes in EPSC before and after bacterial application. As shown in Figure 3a–e, the devices without the LYZ sensing layer exhibited an EPSC of approximately 1500 nA. After the introduction of bacteria, the EPSC decreased to below 250 nA for both Gram-negative and Gram-positive strains, indicating significant suppression of the synaptic current in the device.

However, when the LYZ sensing layer was added to the PTCDI-C_13_, a distinct behavior was observed. As shown in Figure 3f–h, when Gram-negative bacteria were applied, the synaptic current dropped to about 200 nA. In contrast, when Gram-positive bacteria were introduced, the synaptic current significantly increased to approximately 600 nA, indicating a marked difference in response to the different bacterial types.

**Figure 4:**
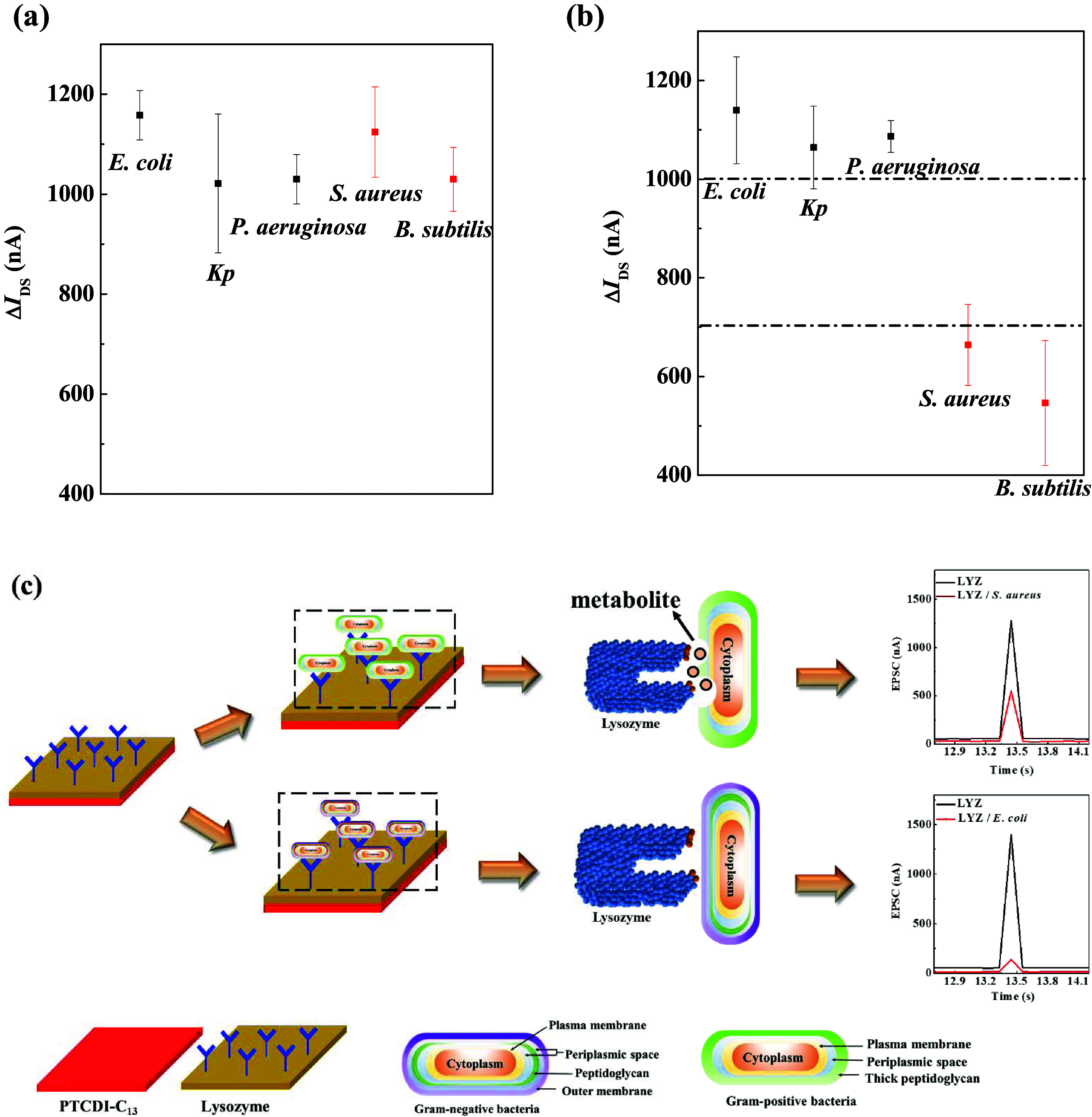
Changes in Δ*I*_DS_ before and after the injection of different bacteria. (a) device without the LYZ sensing layer; (b) device with the LYZ sensing layer; (c) Working mechanisms of differential postsynaptic currents between Gram-negative and Gram-positive bacteria.

To quantify these changes, we calculated the experimental data using the average and standard deviation from more than five devices. The difference between the EPSC peak values before and after bacterial application was defined as Δ*I*_DS_. As shown in Figure 4a,b, when bacteria were applied to PTCDI-C_13_ without the LYZ layer, Δ*I*_DS_ was greater than 1000 nA, suggesting that the device could not differentiate between Gram-negative and Gram-positive bacteria. When the LYZ sensing layer was present, Δ*I*_DS_ remained above 1000 nA for Gram-negative bacteria, indicating minimal impact from the LYZ. However, for Gram-positive bacteria, Δ*I*_DS_ significantly decreased to below 700 nA. This difference may stem from the positive charge of LYZ, which exerts a stronger lytic effect on Gram-positive bacteria. The positive charge of LYZ likely attracts and binds to the negatively charged components of Gram-positive bacteria, leading to cell wall lysis and the subsequent release of captured electrons. This process reduces the presence of Gram-positive bacteria and releases previously trapped carriers back into the channel, thereby increasing the postsynaptic current, as depicted in Figure 4c. We organized the experimental data for the average and standard deviation of Δ*I*_DS_ and included it in Table S1 as Supporting Information.

To further investigate the antibacterial mechanism of the LYZ sensing layer against Gram-positive bacteria, we used AFM to analyze surface morphology. Gram-positive and Gram-negative bacteria were placed on the surfaces of LYZ sensing layers and PTCDI-C_13_ films for comparative analysis. As shown on bottom panel of Figure 1d, when the bacteria contacted the PTCDI-C_13_ film, the AFM images clearly displayed the intact cellular morphology of both Gram-positive and Gram-negative bacteria, indicating that the bacterial structures remained preserved in the absence of LYZ.

In contrast, when the bacteria were placed on the LYZ sensing layer, significant differences emerged. Gram-negative bacteria retained their complete cellular morphology, with their cell wall structures clearly visible. However, for Gram-positive bacteria, the cellular structure was almost entirely degraded, with only the surface morphology of the LYZ sensing layer remaining visible in the AFM images. These observations strongly suggest that LYZ selectively degrades the cell walls of Gram-positive bacteria, which aligns with its role as an *N*-acetylglucosaminidase. LYZ specifically hydrolyzes the peptidoglycan layer in the cell walls of Gram-positive bacteria, leading to structural damage and, ultimately, cell death.

According to existing literature, peptidoglycan is a major component of Gram-positive bacterial cell walls, critical for maintaining cell shape and integrity.^35,36^ These findings are consistent with the results shown in Figures 3 and 4, further validating the antibacterial effect of LYZ and highlighting its potential for applications in antibacterial technologies.

Due to the relatively large size of bacteria, conventional metal electrodes face challenges in forming uniform thin-film coverage through thermal evaporation. Therefore, in capacitor–voltage (*C*–*V*) measurements, we adopted a metal–insulator–metal (MIM) structure, utilizing ion-gel as the dielectric layer and PEDOT:PSS as the electrode material, as shown in Supporting Information Figure S4a. Ion-gel, being a polyelectrolyte material, exhibits capacitance values indicated by the peak in the *C*-*V* curve, as shown in Supporting Information Figure S4b. In the absence of bacteria, using ion-gel as the dielectric layer results in a capacitance of approximately 1 μF/cm^2^. However, upon introducing Gram-negative bacteria (e.g., *E. coli* and *P. aeruginosa*) into the MIM structure, the capacitance significantly increases to approximately 1.5 μF/cm^2^, as shown in Supporting Information Figure S4c and 4d. In contrast, the addition of Gram-positive bacteria (e.g., *S. aureus* and *B. subtilis*) reduces the capacitance to below 1 μF/cm^2^, due to Gram-positive bacteria have the effect of less charge on the surface and increased thickness of dielectric layer, as illustrated in Supporting Information Figure S4e and 4f. This phenomenon could provide a preliminary method for distinguishing bacterial types.

It is important to note that this measurement structure differs from the original device structure, and bacteria cannot be distinctly categorized as either a semiconductor layer or a dielectric layer. Therefore, *C*-*V* measurements alone cannot directly elucidate the detailed mechanisms underlying the sensing process.

### Real-Time Monitoring of Bacterial Detection Using LYZ-Modified OFETs

The previous section highlights the impressive ability of the LYZ sensing layer’s EPSC to differentiate between Gram-negative and Gram-positive bacteria with exceptional resolution. Using a multispike measurement method (On Time = 50 ms, Off Time = 5 ms, Base Voltage = 2 V), we conducted real-time monitoring of *E. coli* and *B. subtilis* as test strains, with bacterial concentrations ranging from 10^4^ to 10^8^ CFU/mL. This real-time monitoring approach allows for rapid analysis of changes in postsynaptic current before and after bacterial application, offering valuable insights into the potential applications of this device in bacterial detection.

**Figure 5:**
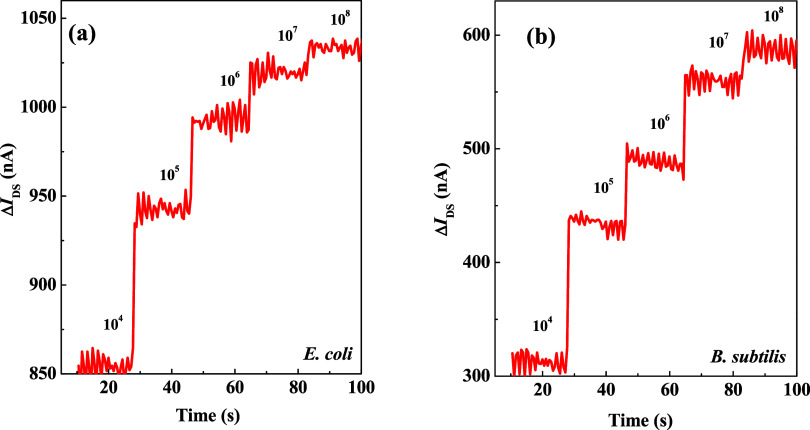
Real-time monitoring results of different bacterial concentrations using multispike measurements, where (a) *E. coli* and (b) *B. subtilis*, displaying their Δ*I*_DS_ variations.

For *E. coli*, the results for Δ*I*_DS_ (shown in Figure 5a) demonstrate an upward trend as bacterial concentration increases, with values ranging from 850 nA to 1050 nA. However, at higher concentrations (10^6^ to 10^8^ CFU/mL), this change stabilizes, likely due to saturation of the effective interaction area between the bacteria and LYZ at 10^6^ CFU/mL. Beyond this point, further increases in bacterial concentration lead primarily to bacterial stacking, which diminishes the device’s detection capability at higher concentrations.

Similarly, for *B. subtilis* (Figure 5b), Δ*I*_DS_ increases from 300 to 600 nA in the 10^4^ to 10^7^ CFU/mL range, confirming the device’s ability to detect Gram-positive bacteria. Notably, even at high concentrations, LYZ effectively lyses the Gram-positive bacteria, maintaining the device’s bacterial detection performance. The experimental results indicate that the device exhibits more pronounced variations at lower bacterial concentrations, underscoring its heightened sensitivity to low-concentration bacterial detection.

This real-time monitoring capability allows for the swift acquisition of data, enabling efficient and accurate bacterial detection. The observed sensitivity, particularly at low bacterial concentrations, positions the LYZ sensing layer-enhanced OFET as a promising tool for applications in rapid bacterial detection, with potential for use in complex biological environments.

## Conclusions

This study demonstrates the superior electrical characteristics of OFETs utilizing BSA as the dielectric layer. By further modifying the surface with a LYZ sensing layer and employing single-spike measurements to analyze EPSC, we successfully differentiated between Gram-negative and Gram-positive bacteria. Real-time monitoring using multispike measurement methods revealed significant variations in Δ*I*_DS_ values depending on the bacterial type. Notably, in the presence of Gram-positive bacteria, the specific lytic activity of LYZ led to smaller changes in Δ*I*_DS_, underscoring its selective action. Our findings highlight the sensor’s excellent sensitivity to low bacterial concentrations, making it a highly effective tool for rapid bacterial detection.

AFM analysis further confirmed the selective degradation of Gram-positive bacterial cell walls by LYZ. The enzymatic activity of LYZ targets Gram-positive bacteria, degrading their cell walls and releasing trapped carriers, which results in distinct changes in Δ*I*_DS_ values. This mechanism enables the reliable differentiation between Gram-positive and Gram-negative bacteria and highlights the device’s biological relevance in bacterial detection. The high sensitivity of the LYZ-modified OFETs to low bacterial concentrations underscores their potential for pathogen discrimination and detection.

Overall, this research advances our understanding of bacterial detection mechanisms and demonstrates the immense potential of LYZ-modified OFETs for developing biosensors capable of real-time microbial pathogen monitoring. These results pave the way for future applications in clinical diagnostics and environmental monitoring, offering new opportunities for rapid and sensitive bacterial detection technologies.

## Supporting Information Available

Table S1: The Δ*I*_DS_ values and standard deviations corresponding to the presence orabsence of the LYZ sensing layer and the injection of different bacteria; Figure S1: The chemical structures and molecular structures of the organic and protein materials; Figure S2: Synaptic current response characteristics of devices with different concentrations of BSA; Figure S3: Transfer curves of devices with different dielectric layers; Figure S4: MIM capacitance measurement (PDF)
