# Supplementary material for: Biocompatible OFETs for Selective and Real-Time Bacterial Detection Using BSA and Lysozyme Layers
Source: ACS Appl Bio Mater. 2025 Apr 3;8(4):2867–74. doi: 10.1021/acsabm.4c01618 (PMC12015955; doi:10.1021/acsabm.4c01618)
Supplement: Supplementary file 1 — mt4c01618_si_001.pdf [file mt4c01618_si_001.pdf]

Biocompatible OFETs for Selective and Real-Time Bacterial Detection Using BSA and Lysozyme Layers

Po-Hsiang Fang[†], Guan-Xu Chen [†], Shuying Wang[‡], Ching-Hao Teng[§], Wen-Chun Huang[§], Horng-Long Cheng[†], Wei-Yang Chou[†, *]

[†] Department of Photonics, National Cheng Kung University, Tainan 70101, Taiwan
[‡] Department of Microbiology and Immunology, Institute of Basic Medical Sciences, College of Medicine, National Cheng Kung University, Tainan 70101, Taiwan
[§] Institute of Molecular Medicine, National Cheng Kung University, Tainan 70101, Taiwan

**Email address: weiyang@ncku.edu.tw*

Table S1. The ΔI_{DS} values and standard deviations corresponding to the presence or absence of the LYZ sensing layer and the injection of different bacteria.

	E. coli	***Kp***	***P. aeruginosa***	***S. aureus***	***B. subtilis***
**ΔI_{DS} without the LYZ sensing layer (nA)**	**1157 ± 49**	**1021 ± 139**	**1029 ± 49**	**1124 ± 90**	**1124 ± 64**
**ΔI_{DS} with the LYZ sensing layer (nA)**	**1140 ± 108**	**1064 ± 84**	**1087 ± 32**	**664 ± 82**	**546 ± 126**

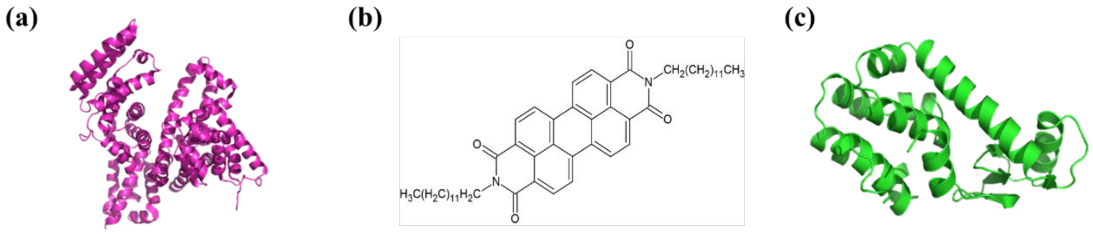

Figure S1. The chemical structures and molecular structures of the organic and protein materials: (a) BSA, (b) PTCDI-C_{13}, and (c) Lysozyme.

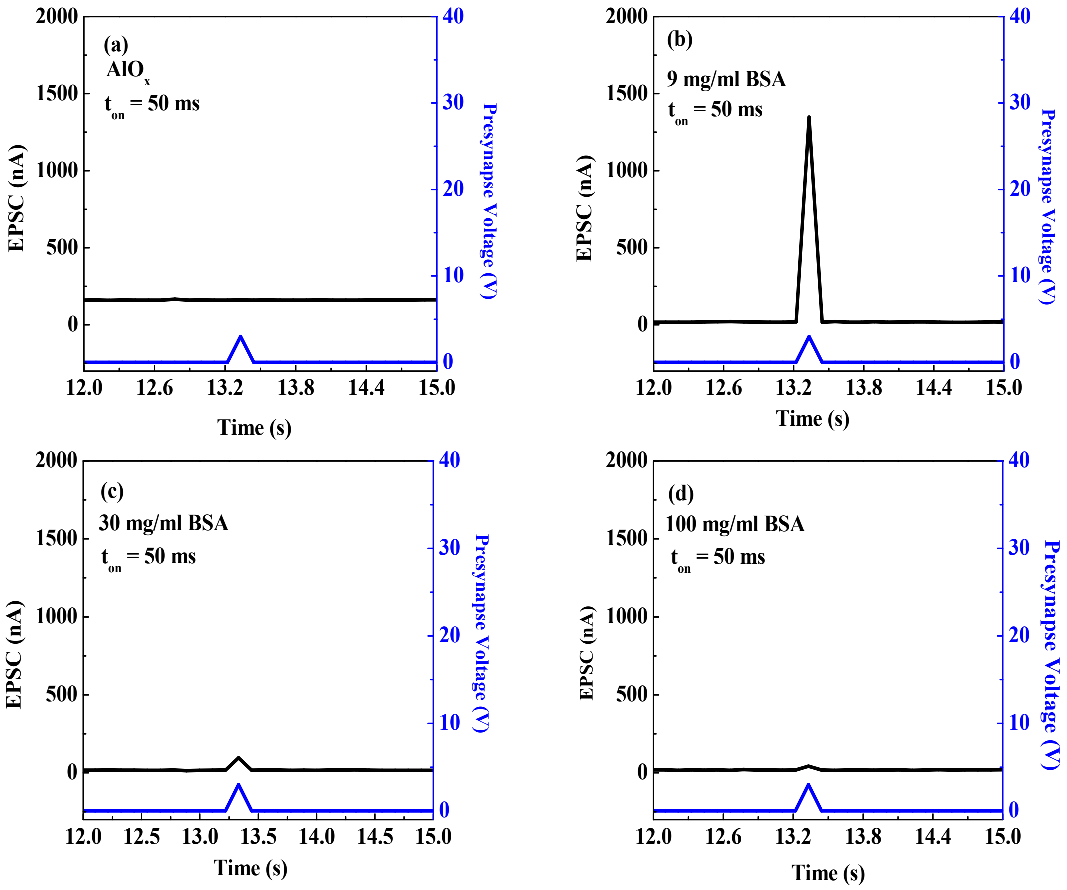

Figure S2. Synaptic current response characteristics of devices with different concentrations of BSA as the dielectric layer: (a) AlO_x, (b) 9 mg/ml BSA, (c) 30 mg/ml BSA, (d) 100 mg/ml BSA.

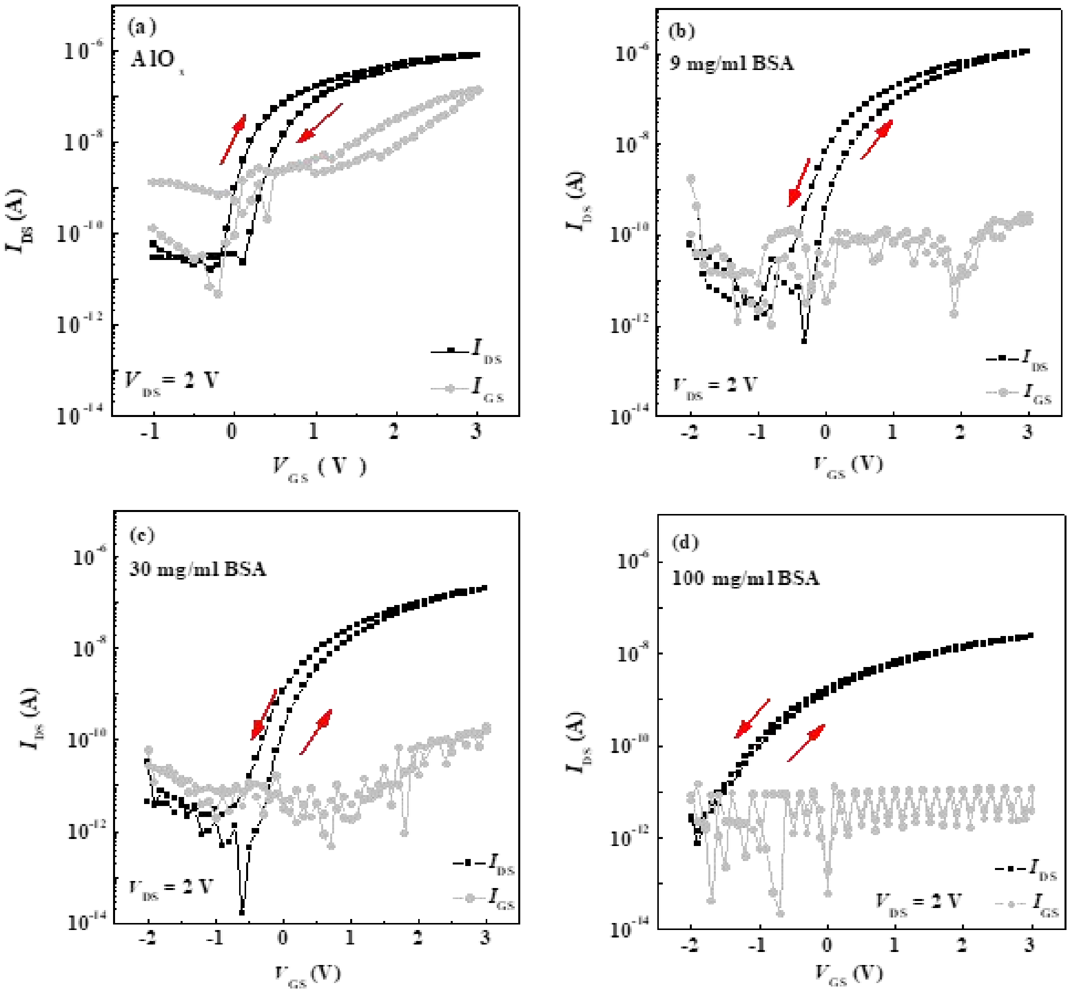

Figure S3. Transfer curves of devices with different dielectric layers: (a) AlO_x, (b) 9.0 mg/mL BSA, (c) 30 mg/mL BSA, and (d) 100 mg/mL BSA.

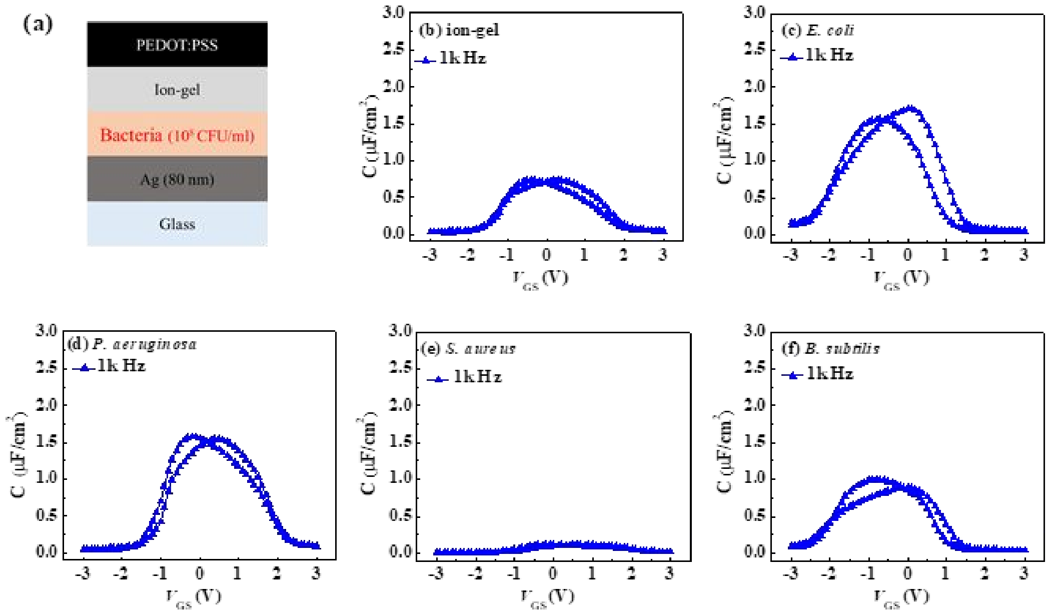

Figure S4. MIM capacitance measurement results with different bacteria added: (a) MIM structure diagram, (b) ion-gel, (c) *E. coli*, (d) *P. aeruginosa*, (e) *S. aureus*, (f) *B. subtilis*.
